# Proteomics Analysis of Lymphoblastoid Cell Lines from Patients with Amyotrophic Lateral Sclerosis

**DOI:** 10.3390/molecules28052014

**Published:** 2023-02-21

**Authors:** Danielle Whitham, Eugene Belenkiy, Costel C. Darie, Aurelian Radu

**Affiliations:** 1 Biochemistry & Proteomics Laboratories, Department of Chemistry & Biomolecular Science, Clarkson University, 8 Clarkson Avenue, Potsdam, NY 13699-5810, USA; 2 Department of Cell, Developmental and Regenerative Biology, Icahn School of Medicine at Mount Sinai, One Gustave L. Levy Place, New York, NY 10029-6574, USA

**Keywords:** amyotrophic lateral sclerosis, proteomics, mass spectrometry, biomarkers

## Abstract

Amyotrophic lateral sclerosis (ALS) consists of the progressive degeneration of motor neurons, caused by poorly understood mechanisms for which there is no cure. Some of the cellular perturbations associated with ALS can be detected in peripheral cells, including lymphocytes from blood. A related cell system that is very suitable for research consists of human lymphoblastoid cell lines (LCLs), which are immortalized lymphocytes. LCLs that can be easily expanded in culture and can be maintained for long periods as stable cultures. We investigated, on a small set of LCLs, if a proteomics analysis using liquid chromatography followed by tandem mass spectrometry reveals proteins that are differentially present in ALS versus healthy controls. We found that individual proteins, the cellular and molecular pathways in which these proteins participate, are detected as differentially present in the ALS samples. Some of these proteins and pathways are already known to be perturbed in ALS, while others are new and present interest for further investigations. These observations suggest that a more detailed proteomics analysis of LCLs, using a larger number of samples, represents a promising approach for investigating ALS mechanisms and to search for therapeutic agents. Proteomics data are available via ProteomeXchange with identifier PXD040240.

## 1. Introduction

Amyotrophic lateral sclerosis (ALS) is a neurodegenerative disease also known as Lou Gehrig’s disease [1,2]. It manifests by the gradual loss of upper and lower motor neurons in the motor cortex, the brain stem nuclei, and the anterior horn of the spinal cord [1,2]. The incidence of ALS is 1–2.6 per 100,000 people per year, the point of prevalence is 3–6 per 100,000 people in Europe and the United States, and the lifetime risk is approx. 1 in 300 [3,4]. The prognosis for survival is 2 to 5 years [3]. There are two types of ALS, differentiated by the genetic background: inherited (familial) and sporadic. The sporadic form represents approx. 90% of all cases [4,5]. The causal factors of sporadic ALS are currently not known. There is no cure or effective treatment for ALS. The treatment consists of multidisciplinary care, including nutritional and respiratory support and symptom management [2,6].

Studying the pathological processes of ALS in the affected cells of alive patients is difficult. The pathological changes in ALS affect the motor neurons, which are the neurons that control the muscles. The death of these neurons leads to general progressive paralysis, which is invariably fatal. Human motor neurons are not accessible for biochemical or cellular investigations, except from deceased patients. Post-mortem neurons display processes that occur in the advanced stage of the disease, which is not very informative about how the pathology is initiated. Moreover, these neurons are affected by the delay between death and the time when the cells become available for analysis. This delay produces damages that make the disease-specific changes more difficult to detect.

As an alternative, model systems are used, based on easily accessible peripheral human cells, either freshly collected or maintained in cell culture. Such systems are used for attempts to discover causal mechanisms and potential therapeutic agents. The relevance of this strategy is supported by numerous observations, which showed that consistent ALS-associated differences exist in peripheral cells unrelated to the nervous systems, e.g., in peripheral blood mononuclear cells or fibroblasts in [7,8].

A particular model system based on blood cells consists of lymphoblastoid cell lines (LCL), which are lymphocytes immortalized using the Epstein–Barr virus [9,10]. Compared to the primary cells, these cells can be stably propagated and multiplied in culture to high numbers and for long periods. This characteristic allows for consistent comparative investigations of various features and interventions, which is favorable for both mechanistic studies and for attempts to find therapeutic agents, e.g., by screening large libraries of small molecules. Most importantly, LCL from ALS donors present clear differences in comparison with non-ALS control cells [11,12].

Genome-wide analyses of differences between ALS and control LCL could reveal cellular networks that are dysregulated in ALS, which could in turn shed light on the mechanisms of the disease and reveal potential biomarkers. Such comparative genome-wide analyses for LCL has been done only at the RNA level and only for the most common variant of familial ALS [13], which represents only 7% of the total ALS cases [4,5].

We present here the comparison of the gene expression at the protein level, for LCL from sporadic ALS vs. control cells from non-diseased donors. 

With few exceptions, the entities that carry out the cellular functions are proteins, not RNAs. However, when attempting to analyze cellular functions, RNA measurements are frequently preferred as surrogates for the abundance of corresponding proteins, although there is only an approximate proportionality between the RNA and the corresponding protein levels. The reason is that the amount of input material necessary for RNA analysis is, as a cell number, almost two orders of magnitude lower for RNA-Seq than for proteomics. Moreover, RNA can be amplified if necessary. A key advantage of using LCL is that the obtainable cell numbers are not limiting factors: the immortalized LCL can be easily multiplied to large numbers in a cell culture, to obtain sufficient material for proteomics studies. 

Most importantly, compared to the RNA level, proteomics can reveal new information about the disease. RNA analysis can leave undiscovered essential pathological differences because RNA could reflect imprecisely variations in some key proteins perturbed in the disease, due, e.g., to differences in the protein synthesis or turnover rates. As shown, for instance, by a recent study on Alzheimer’s disease, RNA-seq analysis missed almost half of the differences detected at the protein level [14].

Proteomics is used not only in the identification of proteins [15,16,17], but also in the identification of protein post-translational modifications (PTMs) [18,19], of stable and transient protein–protein interactions (PPIs) and on the PTMs of these stable and transient PPIs [20,21,22]. In addition, proteomics is used to identify differentially expressed/dysregulated proteins using label-based or label-free proteomics [23,24]. Here, we used label-free proteomics of LCL derived from ALS vs. healthy donors to identify differentially expressed (upregulated and downregulated) proteins that are related to ALS. Some of these proteins, filtered and then validated through a series of statistical analysis and pathway analysis software, confirmed that they are involved in ALS. These and other dysregulated proteins are discussed in relation to previously published studies and the pathogenesis of ALS.

## 2. Results and Discussion

Differentially present proteins. The presence of proteins differentially present in the ALS vs. the healthy donors was investigated using the statistical packages for proteomics: Scaffold, NormalizeR [25], ProDA [26], and ROTS [27].

The Scaffold package includes two tests: Student’s t-test and Fisher’s exact test. Fisher’s exact test performed with the Benjamini–Hochberg adjustment for multiple testing and a threshold of *p* < 0.05 identified 223 proteins that had *p* values below the critical value reported by the test, 0.0178. We excluded from this list the proteins that had less than two non-zero value in the eight samples, and the proteins that showed a difference between the average of ALS and the control sets of less than 150%, corresponding to a log2 ratio smaller than +/− 0.854. Under these conditions, 120 proteins were differentially present, of which 52 were downregulated in ALS and 68 were upregulated (Table 1).

Some of these proteins have been reported to be dysregulated in ALS. For instance, among the most 20 downregulated proteins, 4 are known to be downregulated in ALS: the drebrin-like protein DBNL [28], the calcyclin-binding protein (CacyBP) [29], and the UV excision repair protein RAD23 homolog B (R23B) [30]. The fructose-bisphosphate aldolase A (ALDO A) is downregulated in rapidly progressing ALS [31]. 

Among the 20 most upregulated proteins in ALS, the trifunctional purine biosynthetic protein adenosine-3 (glycyl-tRNA synthetase (GART) enzyme) has a potential connection with ALS: a dominant mutation in this protein causes a toxic gain-of-function, which causes peripheral neuropathy that principally affects the upper limbs [32].

If the proteins detected by us as differentially expressed are indeed perturbed in ALS, and are not just statistical noise, proteins known to be perturbed in ALS should be much less frequent among the proteins that are not differentially expressed in our samples. This is indeed the case: among 25 proteins that are not differentially expressed (having ratios of ALS/healthy between 0.7 and 1.3), none was reported to have differential expression in ALS. Compared to this frequency (0/25), the frequency of ALS-associated proteins among the downregulated proteins is 4/20 = 20%. The difference is statistically significant: as the test cannot compare zero frequency, we compare the immediate higher frequency, 1/25, with 4/20, which leads to p(Exp-Obs>=3) = 0.007 by the binomial test, two-sided.

Other strategies frequently used for detecting differentially present proteins in proteomics data are based on the statistical package limma (“linear models for microarray data”). Limma was initially developed for DNA microarray analysis and subsequently used extensively for the analysis of RNA-seq data [33]. More recently, limma was applied to the analysis of proteomics data [31,34]. We analyzed our data using three packages based on limma. We submitted to these packages the same set of 120 differentially expressed proteins mentioned previously.

NormalizerDE allows the differential expression analysis of liquid chromatography-mass spectrometry data using the empirical Bayes limma approach [25]. The software was accessed online via a web server. The proteomics data across the eight samples were normalized using the CycLoess procedure available in this package. Subsequently the data were log2 transformed using the option available in the package. The analysis revealed three differentially expressed proteins (adj *p* < 0.1): transaldolase (ALDOA), phospho-ribosyl-formyl-glycinamidine synthase (PFAS), and the ATP synthase, H+ transporting, mitochondrial F1 complex, alpha subunit 1 (ATP5F1A or ATP5A1). Two of these proteins, ALDOA and PFAS, were also detected as significantly differentially present by the Scaffold Fisher’s exact test (Table 1). Among the three differentially expressed proteins, two are known to be downregulated in ALS: ALDOA [24] and ATP5F1A [35].

ROTs (reproducibility-optimized test statistic) is a bioconductor R package, which adjusts a modified *t*-statistic according to the inherent properties of the data and provides a ranking of the features based on the differential expression between the two groups [27]. ROTS has been applied successfully in a range of different studies from transcriptomics to proteomics, showing a competitive performance against other state-of-the-art methods. The file generated by Scaffold was normalized using the R function “normalize.quantiles” from BioConductor. The value 0.3 was added to all values to allow for the application of the log function to the initial zero values and the data were subjected to a log2 transformation, to correct the high skewness of the primary data. One protein was detected as significantly downregulated (FDR < 0.1), transaldolase (ALDOA) (average 36.6 in controls versus 0 in ALS). As mentioned, ALDOA is known to be downregulated in ALS.

proDA (inference of protein differential abundance by probabilistic dropout analysis) is a recently introduced method for identifying differentially abundant proteins in label-free mass spectrometry, which boosts statistical power for small sample sizes by using variance moderation [26]. The method is implemented as an open-source R package [26] as the method requires log2 transformed data, 0.3 was added to all the values. Two proteins are detected as significantly differentially expressed (*p* < 0.1). The first is gamma enolase (ENO2), *p*-adj = 0.0032, average 23.9 in controls vs. 12.5 in ALS. ENO2 is detected as significantly downregulated in ALS by the Fisher’s exact test in Scaffold. The second is HSPC108, downregulated in ALS (0.25 in ALS vs. 2.65 in controls), also detected as differentially expressed in Scaffold by Fisher’s exact test. ENO2 levels are known to be elevated in the cerebrospinal fluid (CSF) of ALS patients [36], in the CSF, and serum in patients with a cervico-thoracic form of ALS and in patients with a disease duration from 1 to 4 years [37].

Pathway analysis. Ingenuity pathway analysis (IPA). The list of differentially expressed proteins (Table 1) was subjected to the QIAGEN IPA package (https://digitalinsights.qiagen.com/IPA [38], accessed on 15 September 2022. The top 20 most statistically enriched pathways are listed in Table 2. Perturbations in 6 of these 20 pathways (35%) have been reported to occur in ALS, as detailed below.

The pathway that presents the most statistically significant enrichment is the “EIF2 pathway”. EIF2α is key factor for the initiation of protein translation. Recent studies suggest that endoplasmic reticulum stress may play a critical role in ALS pathogenesis through an altered regulation of the proteostasis, the cellular pathway-balancing protein synthesis, and degradation. EIF2α is a key factor in this process [39]. In response to proteotoxic stress, EIF2α is phosphorylated by MARK2, which is activated via phosphorylation in human patients with ALS [40]. Our differentially expressed protein set is enriched in components of the “Ran signaling pathway”. Ran is a key effector of nucleocytoplasmic-transport and its deficit could play an important role in the pathology of ALS [40,41,42]. The differential protein set is also enriched in members of the “Protein Ubiquitination pathway”, the disruption of which is a widely accepted factor in ALS [43]. Another enriched pathway is the “Antigen presenting pathway”, which was reported to modulate the age of onset of ALS [44]. The blood levels of the “Interferon-gamma (IFN-γ) pathway” present significant differences in ALS patients [45] and the components of this pathway are enriched in our differential protein set. Another pathway that presents enrichment is the “Actin Cytoskeleton Signaling”, which contributes to motor neuron degeneration [46]. The modulation of actin polymerization affects nucleocytoplasmic transport in multiple forms of ALS [47]. 

Two other pathways associated with neuronal pathology are also in the top 20 of the enriched pathways: “Multiple Sclerosis Signaling Pathway” and “Neuro-inflammation Signaling Pathway”. This observation supports the assumption that although LCLs are peripheral cells, some processes in these cells correlate with processes in neuronal cells, and therefore LCLs can be used to derive information about the pathology of the neuronal cells affected by the disease. 

The last column in Table 2 (“Ratio”) represents the ratio between the proteins that belong to the specified pathway and are differentially present in ALS, and the total number of the proteins in the pathway. The average ratio is 6.7% (range 2.1–21%). These percentages seem low, but it should be taken into account that only approx. 1300 proteins have been detected by us, which represent probably only approx. 10% of the total number of proteins expressed in LCLs. Based on this assumption, it can be extrapolated that if all the proteins would be detected, the “coverage” of the differential pathways would be on average approx. 67%, the majority of the proteins participating in the top 20 pathways.

pathfindR is an R package for the identification of enriched pathways based on protein–protein interaction networks [48]. pathfindR can identify relevant pathways which cannot be identified by other tools [48]. The package can use five protein interaction networks (Biogrid, STRING, GeneMania, IntAct, KEGG) and seven gene sets (GO-CC (cellular component), GO-BP (biological process), GO-MF (molecular function), GO-All (all combined), and KEGG, Biocarta, Reactome). These combinations detect a total number of 223 significantly enriched pathways (*p* < 0.05). The combination Biogrid–Reactome detects the highest number (119) of significantly enriched pathways. 

The most frequent significantly enriched pathways are related to rRNA, ribosomes, protein translation (15 occurrences), antigen presentation (9), protein ubiquitination–protein degradation (6), and apoptosis regulation (9). Some of these perturbations have been detected previously in ALS. Relevant for the first category, the following perturbations in ALS has been reported: a profound destabilization of ribosomal and mitochondrial RNAs [49], perturbation in the ribosomal function [50], and increased ribosome numbers in axons as an early event [51]. Regarding the second category, protein homeostasis, ALS is associated with proteostasis collapse [43]. Central to the maintenance of proteostasis are the predominant protein degradation pathways, the ubiquitin–proteasome system (UPS), and the autophagy system.

Gene set enrichment analysis (GSEA) is a computational method that determines whether a set of genes shows statistically significant similarity with gene sets from a large collection that have certain characteristics, e.g., participate in specific cellular or molecular processes, or are perturbed in diseases or by various interventions, e.g., specific gene modifications or treatment with chemical agents [52]. GSEA was initially introduced for analysis of cDNA microarrays and was subsequently adapted for RNA-Seq data. Based on the correlation between the RNA and protein levels, the method could also provide information about protein sets, as illustrated below. Compared to other pathway identification methods, many of the gene sets available in GSEA sets are not pieced together from interactions between protein pairs or protein subsets but are the direct results of RNA-seq or microarray experiments, with no theoretical deductions regarding the pathway that may or may not be actually functioning in cells. 

**Figure 1:**
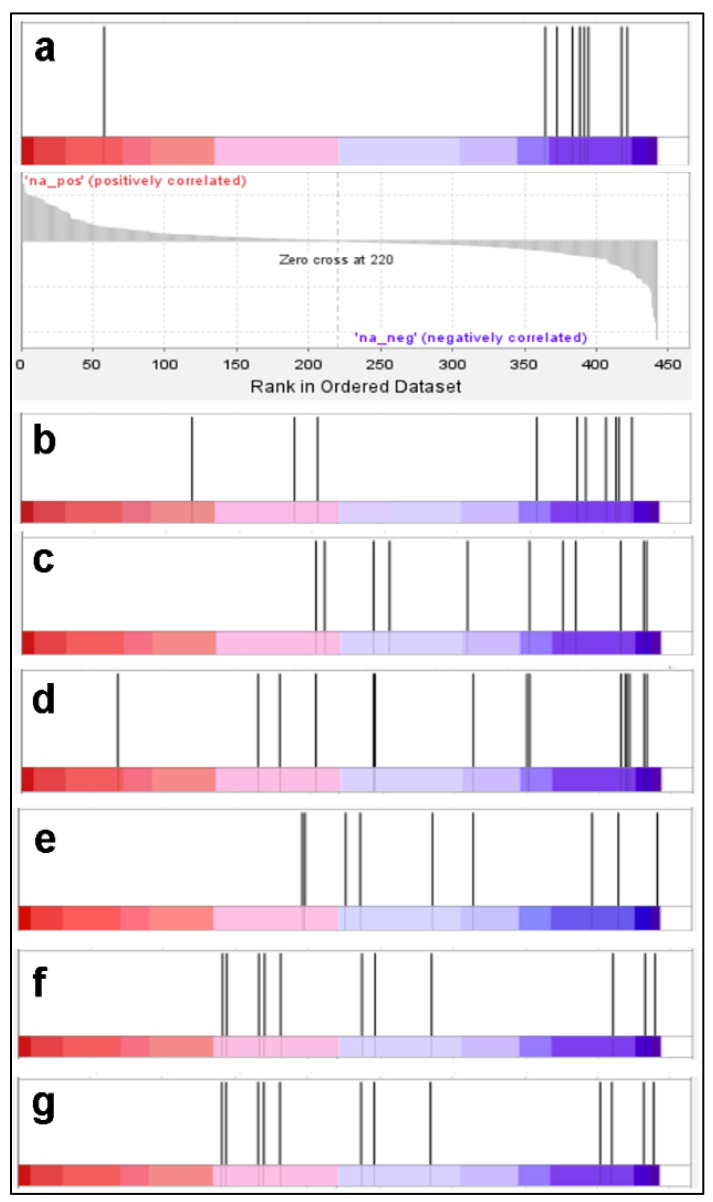
Proteins downregulated in the ALS samples are enriched in proteins of GSEA sets known to be perturbed in ALS: (**a**) the “E2F1_UP.V1_UP” set. The lower histogram shows the ratio in ALS vs. control for each protein differentially present in our samples, ranked from the most upregulated (on the left) to the most downregulated (to the right). The vertical bars in the upper panel show the positions of the proteins from the GSEA set present in the proteomics set; (**b**–**g**): similar upper histograms for other GSEA pathways enriched in ALS differentially expressed proteins; (**b**) “Rutella response to HGF_DN” (genes induced by HGF); (**c**) “KEGG spliceosome”; (**d**) “module 183 cancer RNA splicing”; (**e**) “QI_Hypoxia”, (HIF-induced genes); (**f**) “GOBP positive regulation of protein localization to nucleus”; (**g**) “GOBP regulation of protein localization to nucleus”.

From the list of 614 proteins present in at least 2 samples, as generated by Scaffold and having associated NCBI gi tags, GSEA identified 484 proteins. The parameters used for analysis were: 1000 permutations, no collapse (i.e., data are used in the original format), and enrichment statistics-weighted, max size 500, min size 8, normalization mode: none. Eight types of cumulative gene sets are available in GSEA for comparison with the submitted set. The significance threshold used was *p* < 0.05 and the FDR (false discovery rate) was 0.25. Across all 8 sets, GSEA identifies 147 sets that are significantly enriched in genes present in our set of ALS differentially expressed genes. Six of these are clearly relevant for ALS, based on published information about processes perturbed in ALS, or interventions that could alleviate ALS. These enrichments are shown in Figure 1). 

(a) “E2F1_UP.V1_UP” set, which contains genes up-regulated by everolimus. Everolimus, an inhibitor of the TORC pathway, is a derivative of rapamycin, which is an mTOR inhibitor. This signaling pathway is upregulated in ALS and reduces autophagy. Rapamycin has been evaluated for ALS therapy by the restoration of autophagy [53].

(b) “Rutella response to HGF_DN”, this set contains genes induced by HGF (hepatocyte growth factor), which is currently explored as a potential therapy for ALS [54]. The implication is that the LCLs from ALS patients present a reduction in proteins whose restoration by HGF could treat ALS.

(c) and (d) “KEGG spliceosome” and “module 183 cancer RNA splicing”. The RNA splicing process is known to be defective in ALS [55].

(e) “QI_Hypoxia”, which contains HIF-induced genes, as detected by applying hypoxia to a prostate cancer cell line. Deficiency in HIF1-alpha signaling is known to occur in ALS, due to defective import of HIF1-alpha from cytoplasm in to the nucleus, which is necessary to trigger the induction of hypoxia-responsive genes [56,57].

(f) and (g) “GOBP positive regulation of protein localization to nucleus” and “GOBP regulation of protein localization to nucleus”; there is ample evidence that nucleocytoplasmic-transport deficits could play an important role in the pathology of ALS [40].

In all the above cases, the enrichment occurs for genes that are downregulated in the ALS LCLs, and the direction of the perturbation is consistent with the pathological significance of the GSEA pathways. This observation further indicates that the perturbations detected in LCLs in our proteomics study correctly reflect the pathological processes that occur in ALS in human patients. 

An interesting observation is that the proteins upregulated in our data do not appear to belong to any functional pathways. The explanation could be that the common feature of these proteins is not participation in some biological pathways, but common physical characteristics. For instance, these proteins could be more prone to folding errors, or more resistant to degradation by the unfolding protein response machinery, leading to the accumulation of these proteins, which confusingly appearing to be upregulated. Notably, such defect cannot be detected by RNA-seq or microarray studies, but can be revealed by proteomics, which detects protein fragments and does not depend on the folding state of the proteins.

In conclusion, the proteomics analysis LCLs derived from ALS donors versus controls revealed statistically significant differences in some proteins and pathways. A subset of these pathways has been previously reported to be perturbed in ALS, while others have not been previously linked to ALS. The latter observation suggests that proteomics of LCLs from ALS donors versus controls could be a useful strategy to derive new information about the ALS pathological processes and identify new markers that could be used for drug discovery.

Compared to primary cells freshly obtained from donors, LCLs offer a stable platform, which can be used repeatedly over long periods, to compare the effects of various interventions. Moreover, the cells can be expanded to large numbers, sufficient for drug discovery by high content screening of thousands of small compounds, or for genome-wide gene knockout of overexpression.

Apart from the statistical significance included in the software packages analyzed, the fact that proteins known to be perturbed in ALS are significantly more frequent among the proteins detected by the proteomics study to be differential, which increases the confidence that the proteomics analysis, although performed on a small number of samples, indeed detects proteins differentially expressed in ALS.

A new hypothesis supported by this study is that many proteins upregulated in ALS do not belong to specific upregulated pathways, but rather have similarities in either a higher propensity of failing to fold properly, or, if unfolded, they have a higher resistance to degradation by the unfolded protein response (UPR) system. Investigations aimed at finding such common characteristics, such as common motifs in the protein structures, could shed a new mechanistic light on ALS, and could suggest new therapies.

Overall, the study yielded promising results, which supports the utility of LCLs for ALS studies, and, in particular, the proteomics approach. Further studies using higher numbers of samples and other proteomics methods could be used to confirm and extend the reported observations.

## 3. Materials and Methods

### Cell Lines

ALS and control lymphoblastoid cell lines (B-lymphocytes immortalized with the Epstein–Barr virus) [9,10] were obtained from NINDS Repository at Coriell Cell Biorepositories, Camden, New Jersey. In total, 4 cell lines originated from ALS donors (2 females and 2 males, 44 years old) and 4 lines were derived from healthy donors (2 females and 2 males, 41 to 47 years old). The cells were maintained in T25 flasks in a vertical position in 10 mL of RPMI 1640 medium containing 10% heat-inactivated fetal bovine serum (FBS), penicillin/streptomycin at 100 U/mL and 100 μg/mL in an incubator in 5% CO_2_ at 37 °C.

Protein extraction. Each cell line was grown to 500,000 cells/mL in 2 T25 flasks with 10 mL of medium. The proteins from FBS were removed by placing the cells from each flask in 50 mL conical tubes, centrifugation at 1410 RPM for 10 min at 4 °C, followed by resuspension of the pellets in 40 mL of washing buffer (0.29 M mannitol, 10 mM Tris pH 7.4). The centrifugations were repeated 3 times. The last pellet was resuspended in 1 mL of cold lysis buffer (1 M NaOH) after adding to the pellet 10 µL of Protease and Phosphatase Inhibitors Cocktail (MSSAFE-1VL, Sigma Aldrich St. Louis, MO, USA)). The pellet was resuspended by repeated pipetting and incubated on ice for 30 min, followed by microfugation at 14,000 RPM for 20 min at 4 °C. The supernatant was collected and the protein concentration was measured using the BCA colorimetric assay kit (Pierce Cat. No. 23227). Samples of 250–300 ug of protein were flash-frozen in liquid nitrogen and shipped for mass spectrometry analysis.

Mass spectrometry. Sample pre-processing. The samples were subjected to desalting and buffer exchange using Amicon Ultra 0.5 mL 3 kDa centrifugal filters (Millipore, Burlington, MA, USA), as previously described [58]. The total volume was brought to 200 µL using HPLC-grade water and centrifuged for 10 min at 13,000 RPM. HPLC-grade water (100 µL) was added to each filter and centrifuged for another 5 min at 13,000 RPM. The volume left in the filter (70–100 µL) was pipetted repeatedly up and down. The filter was then placed upside down in a new 1.5 mL tube and centrifuged for 2 min at 3500 RPM. The proteins left in the filter were collected by placing 100 µL of HPLC water in the filter and repeating the transfer to the new 1.5 mL tube. The protein concentration was then measured using a Bradford assay. Volumes of solution containing 200 ug of protein were dried down completely in a SpeedVac and resuspended in 20 µL of 6 M urea, 100 mM of Tris buffer pH 7.8, and sonicated for 30 min. Each sample was reduced by adding 1 µL of a solution containing 200 mM of dithiothreitol (DTT) and 100 mM of Tris buffer, gently vortexed and incubated at room temperature (RT) for one hour. Subsequently, the samples were alkylated using 4 µL of the alkylating agent, 200 mM of iodoacetamide (IAA), and 100 mM of Tris buffer, then gently vortexed and incubated in the dark for an hour at RT. Each sample was reduced again by adding 4 µL of the same reducing agent as before. HPLC water (155 µL) was added to each sample to reduce the urea concentration and then 20 ug trypsin in 100 µL of 20 mM Tris was added and the samples were incubated for 16–18 h at 37 °C. The reaction was stopped by adding a drop to glacial acetic acid to bring the pH < 6, and the samples were dried completely in a SpeedVac. The dried samples are then resolubilized in 100 µL of 0.1% formic acid and sonicated for 15 min. For each sample, a volume of 50 µL of 50% acetonitrile and 0.1% formic acid was pushed through 1 mg top-tips (Glygen) 3 times into waste, followed by 3 washes using 0.1% formic acid. The samples were added to the top-tips, pushed into the same 1.5 mL tube, reapplied to the same top-tip, and pushed into the same tube. Then, 50 µL of 0.1% formic acid was pushed through the tip into waste twice. After that, 50 µL of elution buffer (50% acetonitrile with 0.1% formic acid) was added to the tip and pushed into a new 0.6 mL tube. The same 50 µL volume was then pushed through the tip a second time. A new volume of 50 µL elution buffer was added to the tip and pushed into the same 0.6 mL tube, and then pushed again through the tip. The total volume in the new 0.6 mL tube was 100 µL. The samples were then dried down completely and re-solubilized in 50 µL 2% acetonitrile and 0.1% formic acid.

Proteomics analysis. The samples were analyzed using a NanoAcquity UPLC coupled with a QTOF Xevo G2-XS mass spectrometer (both from Waters Corp, Milford, MA, USA). The peptides were loaded on a NanoAcquity BEH130 C18 1.7 µm reversed phase chromatography UPLC column (Waters, Milford, MA, USA), that was coupled to a fused silica nano-ESI emitter (363 µm OD × 20 µm ID × 6.25 cm length, New Objective, Littleton, MA, USA). The samples (3 µL, 12 µg protein) were injected onto the column followed by a linear gradient with a flow rate of 0.6 µL/min: 1–7% organic solvent B (acetonitrile with 0.1% formic acid) for 1–9 min, 7% B (9–15 min), 7–40% B (15–350 min), 40% B (350–359 min), and back to the initial conditions of 1% B (359–360). Each sample was followed by 3 20 min washes, 2 30 min washes, 1 130 min wash, and 1 35 min external standard Glu-Fib. The MS instrument parameters were: capillary voltage: 2.8 kV, sampling cone: 40 V, source temperature: 80 °C, nano flow gas: 0.30 bar, purge gas: 50 L/h, gas source: nitrogen generator (Peak Scientific, Inchinnan, UK). The acquisition mode is positive polarity in sensitivity mode. MS survey scans from *m*/*z* of 100–2000 and automatic data-dependent analysis (DDA) of the top 8 ions with a high intensity with charges of 2+ to 6+. The MS/MS acquisition was then triggered when the MS signal rose above 1000 counts/second. The top 8 ions selected for MS/MS from the MS survey were subjected to further fragmentation until the MS/MS ion count reached 10,000/sec or after 0.4 s have elapsed. The charge state recognition was used for collision energy with a collision gas being argon. The washing runs (20 min runs for short washing steps and 130 min runs for long, extensive washing steps) were acquired under MSe conditions scanning a range *m*/*z* of 50–2000. Each of the 4 ALS and 4 control samples were run in triplicate.

Protein identification. The raw data were submitted ProteoWizard MS Convert to convert the input raw data to mzML readable data, and then the mzML data were further submitted to our in-house Mascot Daemon server (Matrix Science, London, UK) for database search. The following parameters were used: NCBI database (Homo sapiens) with a peptide tolerance of 1.3 Da and zero 13C, a MS/MS tolerance of 0.8 Da, trypsin enzyme, 3 maximum missed cleavages, a fixed modification of carbamidomethyl on a cysteine and a variable modification of oxidation on methionine. 

The output of the database search (*.dat* files) were further analyzed by the software package Scaffold v. 4.3.4 (Proteome Software, Portland, OR, USA) for protein identification, label-free quantitation, and statistical analysis. The data files were then loaded and analyzed with the following options: protein threshold 90%, minimum number of peptides = 1; peptide threshold 80%; total spectrum count, no filter. The threshold for the minimum number of peptides was selected as 1, to avoid missing true positives. The caveat is obviously that the number of false positives is higher than for more stringent thresholds. 

To determine the integrity of the data and proteins identified, we narrowed the thresholds for protein identification. The threshold used in this publication was a peptide threshold of 90%, minimum number of peptides as 1, and a peptide threshold of 80%. This had an FDR of 1.3% for proteins, and an FDR of 2.68% for spectra. To narrow this, we increased both the protein and peptide threshold as well as increased the minimum number of peptides identified. We did 3 different trials, each with a narrower threshold. The thresholds used were as follows: protein threshold of 90, min peptide of 1, and peptide threshold of 90 (90-1-90), a second with protein threshold of 90, min peptide of 2, and peptide threshold of 90 (90-2-90), and, finally, a protein threshold of 90, min peptide of 2, and peptide threshold of 95 (90-2-95). The first variation, 90-1-90, had an FDR of 1.3% for proteins and an FDR of 1.49% for spectra. The second, 90-2-90, had an FDR for proteins of 0.4% and an FDR for spectra of 1.46%. The final trial, 90-2-95, had and FDR of 0.3% for proteins and an FDR of 0.82% for spectra. When narrowed, we were able to identify almost all of the proteins in our tightest constraint, and others were still found, but were considered to be insignificant (*p*-value ≤ 0.05) under the specific conditions. Doing this confirmed that the proteins identified in this study are most likely true positives. 

Proteins differentially expressed between the ALS and the control cells were assessed using Scaffold and the software packages NormalizeR [25], ProDA [26], and ROTS [27]. 

Pathway analysis was performed using the software packages Ingenuity [38], PathfindR [48], and GSEA [52].

## Figures and Tables

**Table 1:** Dysregulated proteins identified in our study.

	Protein Names	Accession Number	Gene Symbol	*p*-Value	Av Control	Av ALS	Av Control/AV ALS
1	Beta actin variant	62897625		<0.00010	187.8	0.0	inf
2	Fructose-bisphosphate aldolase A (EC 4.1.2.13) (lung cancer antigen NY-LU-1) (muscle-type aldolase)	16740581	ALDOA ALDA	<0.00010	45.8	0.0	inf
3	MHC class I antigen	302144416	HLA-A	<0.00010	30.0	0.0	inf
4	T-complex protein 1 subunit theta (CCT-theta)	544711070	CCT8 hCG_1810843	<0.00010	15.5	0.0	inf
5	MHC class II antigen	193227846	HLA-DRB1	<0.00010	4.8	0.0	inf
6	HLA class II histocompatibility antigen, DR alpha chain (MHC class II antigen DRA)	52426774	HLA-DRA HLA-DRA1	<0.00010	3.5	0.0	inf
7	phosphoribosylaminoimidazole carboxylase, phosphoribosylaminoimidazole succinocarboxamide synthetase, isoform CRA_a	119625894	PAICS	0.00036	2.8	0.0	inf
8	cDNA FLJ54071, highly similar to drebrin-like protein	194383996		0.00075	2.5	0.0	inf
9	Phosphopyruvate hydratase (EC 4.2.1.11) (2-phospho-D-glycerate hydro-lyase)	119590453	EDARADD hCG_1640319	0.00075	2.5	0.0	inf
10	Serine/arginine-rich splicing factor 2 (Splicing component, 35 kDa) (splicing factor SC35) (splicing factor, arginine/serine-rich 2)	119609849	SFRS2 hCG_27842	0.0015	2.3	0.0	inf
11	Ubiquitin-conjugating enzyme E2 variant 1 (UEV-1) (CROC-1) (TRAF6-regulated IKK activator 1 beta Uev1A)	73536113	UBE2V1 CROC1 UBE2V UEV1 P/OKcl.19	0.0032	2.0	0.0	inf
12	Hematopoietic cell-specific Lyn substrate 1, isoform CRA_a	119599911	HCSL1	0.0032	2.0	0.0	inf
13	Small nuclear ribonucleoprotein Sm D2 (Sm-D2) (snRNP core protein D2)	4759158	SNRPD2 SNRPD1	0.0065	1.8	0.0	inf
14	Transcription factor BTF3 (nascent polypeptide-associated complex subunit beta) (NAC-beta) (RNA polymerase B transcription factor 3)	20070130	BTF3 NACB OK/SW-cl.8	0.013	1.5	0.0	inf
15	Calcyclin-binding protein (CacyBP) (hCacyBP) (S100A6-binding protein) (Siah-interacting protein)	193787507	CACYBP S100A6BP SIP PNAS-107	0.013	1.5	0.0	inf
16	Vasodilator-stimulated phosphoprotein isoform X1	530417131	VASP	0.013	1.5	0.0	inf
17	UV excision repair protein RAD23 homolog B (HR23B) (hHR23B) (XP-C repair-complementing complex 58 kDa protein) (p58)	18089249	RAD23B	0.0046	2.5	0.3	10.00
18	MHC class II antigen	32401120	HLA-DQA1/DRA	<0.00010	4.8	0.5	9.50
19	cDNA FLJ77316, highly similar to Homo sapiens interferon, gamma-inducible protein 30 (IFI30), mRNA	158256534		0.00013	4.5	0.5	9.00
20	Proteasome subunit alpha type-2	21465643	PSMA2	0.00024	4.3	0.5	8.50
21	Suppression of tumorigenicity 13 (colon carcinoma) (Hsp70 interacting protein), isoform CRA_b	119580799	ST13	<0.00010	5.3	0.8	7.00
22	Heterogeneous nuclear ribonucleoprotein A1 (Helix-destabilizing protein) (hnRNP core protein A1)	194389862		0.0015	3.5	0.5	7.00
23	Stomatin-like protein 2, mitochondrial (SLP-2) (EPB72-like protein 2) (paraprotein target 7) (paratarg-7)	6841440	STOML2 SLP2 HSPC108	0.0049	3.0	0.5	6.00
24	Transferrin receptor (p90, CD71), isoform CRA_b	119574056	TFRC	0.0087	2.8	0.5	5.50
25	MHC class I antigen	161376703	HLA-A	<0.00010	30.3	5.8	5.26
26	Transaldolase (EC 2.2.1.2)	48257056	TALDO1 TAL TALDO TALDOR	0.00016	6.0	1.3	4.80
27	Transforming protein RhoA (EC 3.6.5.2) (Rho cDNA clone 12) (h12)	21466025	RHOA ARH12 ARHA RHO12	0.0013	5.0	1.3	4.00
28	Eukaryotic translation initiation factor 4H (eIF-4H) (Williams–Beuren syndrome chromosomal region 1 protein)	11559923	EIF4H KIAA0038 WBSCR1 WSCR1	0.012	3.5	1.0	3.50
29	Signal recognition particle 9 kDa protein (SRP9)	11513832	SRP9	0.0097	4.0	1.3	3.20
30	Retinoic acid receptor alpha (RAR-alpha) (nuclear receptor subfamily 1 group B member 1)	1314308	RARA NR1B1	<0.00010	27.8	8.8	3.17
31	Acetyl-CoA acetyltransferase, cytosolic (EC 2.3.1.9) (acetyl-CoA transferase-like protein) (cytosolic acetoacetyl-CoA thiolase)	546901	ACAT2 ACTL	0.0014	6.3	2.0	3.13
32	SERPINE1 mRNA-binding protein 1, isoform CRA_	119626894	SERBP1	0.015	3.8	1.3	3.00
33	cDNA, FLJ94551	189054116		0.0066	5.0	1.8	2.86
34	cDNA FLJ53392, highly similar to ubiquitin-activating enzyme E1	194384538		0.00016	10.3	3.8	2.73
35	Peroxiredoxin-5, mitochondrial (EC 1.11.1.24) (Alu corepressor 1) (antioxidant enzyme B166) (AOEB166) (liver tissue 2D-page spot 71B) (PLP) (peroxiredoxin V) (Prx-V) (peroxisomal antioxidant enzyme) (TPx type VI) (thioredoxin peroxidase PMP20) (thioredoxin-dependent peroxiredoxin 5)	15826629	PRDX5 ACR1 SBBI10	0.00046	9.3	3.5	2.64
36	MHC class I antigen	572455386	HLA-B	<0.00010	19.0	7.3	2.62
37	PREDICTED: cofilin-1 isoform X2 [Nomascus leucogenys]	332250232		<0.00010	29.8	11.5	2.59
38	Endoplasmic reticulum resident protein 29 (ERp29) (endoplasmic reticulum resident protein 28) (ERp28) (endoplasmic reticulum resident protein 31) (ERp31)	192987144	ERP29 C12orf8 ERP28	0.0013	9.0	3.8	2.40
39	ATP-binding cassette sub-family E member 1 (2′-5′-oligoadenylate-binding protein) (HuHP68) (RNase L inhibitor) (ribonuclease 4 inhibitor) (RNS4I)	987870	ABCE1 RLI RNASEL1 RNASELI RNS4I OK/SW-cl.40	0.0095	6.3	2.8	2.27
40	Ubiquitin-like protein ISG15 (interferon-induced 15 kDa protein) (interferon-induced 17 kDa protein) (IP17) (ubiquitin cross-reactive protein) (hUCRP)	4826774	ISG15 G1P2 UCRP	0.0095	6.3	2.8	2.27
41	Quinone oxidoreductase (EC 1.6.5.5) (NADPH:quinone reductase) (zeta-crystallin)	13236495	CRYZ	0.017	5.5	2.5	2.20
42	Proteasome subunit alpha type-4 (macropain subunit C9) (multicatalytic endopeptidase complex subunit C9) (proteasome component C9) (proteasome subunit L)	34783332	PSMA4 HC9 PSC9	0.0031	8.8	4.0	2.19
43	MHC class I antigen	221148541	HLA-A	<0.00010	25.5	11.8	2.17
44	Proteasome subunit alpha type-1 (30 kDa prosomal protein) (PROS-30) (macropain subunit C2) (multicatalytic endopeptidase complex subunit C2) (proteasome component C2) (proteasome nu chain)	13543551	PSMA1 HC2 NU PROS30 PSC2	0.0036	9.0	4.3	2.12
45	ADP-ribosylation factor 3	119626762	ARF3	0.008	7.8	3.8	2.07
46	ADP-ribosylation factor	4502203	ARF3 hCG_40390	0.0064	8.3	4.0	2.06
47	Protein disulfide-isomerase A3 (EC 5.3.4.1) (58 kDa glucose-regulated protein) (58 kDa microsomal protein) (p58) (disulfide isomerase ER-60) (endoplasmic reticulum resident protein 57) (ER protein 57) (ERp57) (endoplasmic reticulum resident protein 60) (ER protein 60) (ERp60)	114793397	PDIA3 ERP57 ERP60 GRP58	0.00015	17.5	8.8	2.00
48	MHC class I antigen	255682810	HLA-B	0.0046	9.5	4.8	2.00
49	10 kDa heat shock protein, mitochondrial (Hsp10) (10 kDa chaperonin) (chaperonin 10) (CPN10) (early pregnancy factor) (EPF)	4008131	HSPE1	<0.00010	33.8	18.3	1.85
50	Gamma-enolase (EC 4.2.1.11) (2-phospho-D-glycerate hydro-lyase) (enolase 2) (neural enolase) (neuron-specific enolase) (NSE)	182118	ENO2	<0.00010	24.0	13.0	1.85
51	Thioredoxin (Trx) (ATL-derived factor) (ADF) (surface-associated sulfhydryl protein) (SASP) (allergen Hom s Trx)	685425705	TXN TRDX TRX TRX1	0.0036	12.0	6.5	1.85
52	Adenylyl cyclase-associated protein	5453595	CAP1 hCG_2033246	<0.00010	34.0	18.8	1.81
53	Eukaryotic translation initiation factor 5A-1 (eIF-5A-1) (eIF-5A1) (eukaryotic initiation factor 5A isoform 1) (eIF-5A) (Rev-binding factor) (eIF-4D)	4503545	EIF5A	0.007	8.3	15.0	0.55
54	Tubulin beta-6 chain (tubulin beta class V)	14210536	TUBB6	0.00014	14.5	27.5	0.53
55	60S ribosomal protein L36 (large ribosomal subunit protein eL36)	16117794	RPL36	0.0042	7.0	13.8	0.51
56	14-3-3 protein gamma (protein kinase C inhibitor protein 1) (KCIP-1) [cleaved into: 14-3-3 protein gamma, N-terminally processed]	380764684	YWHAG	<0.00010	18.3	35.0	0.52
57	14-3-3 protein zeta/delta (protein kinase C inhibitor protein 1) (KCIP-1)	347948616	YWHAZ	<0.00010	25.3	51.8	0.49
58	hCG2038942, partial	119602344		<0.00010	18.8	40.8	0.46
59	Elongation factor 1-gamma (EF-1-gamma) (eEF-1B gamma)	15530265	EEF1G EF1G PRO1608	<0.00010	9.0	20.8	0.43
60	HLA class II histocompatibility antigen gamma chain (HLA-DR antigens-associated invariant chain) (Ia antigen-associated invariant chain) (Ii) (CD antigen CD74) [cleaved into: class-II-associated invariant chain peptide (CLIP)]	32132	CD74 DHLAG	<0.00010	7.5	17.8	0.42
61	60S ribosomal protein L23a (Large ribosomal subunit protein uL23)	17105394	RPL23A	<0.00010	7.8	20.0	0.39
62	MHC class I antigen	388240742	HLA-B	<0.00010	8.0	20.8	0.39
63	MHC class I antigen	563403983	HLA-C	<0.00010	9.3	24.5	0.38
64	60S ribosomal protein L8 (large ribosomal subunit protein uL2)	15082586	RPL8	0.01	2.3	6.0	0.38
65	Tyrosine-protein phosphatase non-receptor type 6 (EC 3.1.3.48) (hematopoietic cell protein-tyrosine phosphatase) (protein-tyrosine phosphatase 1C) (PTP-1C) (protein-tyrosine phosphatase SHP-1) (SH-PTP1)	18104989	PTPN6 HCP PTP1C	0.0006	4.0	10.8	0.37
66	Ribosomal protein L7a, isoform CRA_	119608467		0.0022	3.0	8.3	0.36
67	Fermitin family homolog 3 (Kindlin-3) (MIG2-like protein) (Unc-112-related protein 2)	28626504	FERMT3 KIND3 MIG2B URP2	0.014	1.8	5.0	0.35
68	40S ribosomal protein S6 (phosphoprotein NP33) (small ribosomal subunit protein eS6)	337514	RPS6 OK/SW-cl.2	<0.00010	8.0	23.0	0.35
69	Ig alpha-2 chain C region	70058	IGHA2	0.001	3.0	8.8	0.34
70	cDNA, FLJ93036, highly similar to Homo sapiens tyrosine 3-monooxygenase/tryptophan 5-monooxygenaseactivation protein, eta polypeptide (YWHAH), mRNA	189069195		0.00015	3.8	11.3	0.33
71	5C5	3868714		0.0013	2.5	7.8	0.32
72	Ig M Fc	222978		0.0036	2.0	6.3	0.32
73	MHC class I antigen	572455377	HLA-B	<0.00010	12.0	38.0	0.32
74	Actin-like protein	62421128	ACT	<0.00010	16.8	53.5	0.31
75	Beta-actin-like protein 2 (kappa-actin)	62420949	ACTBL2	<0.00010	47.3	166.3	0.28
76	60S ribosomal protein L24 (60S ribosomal protein L30) (large ribosomal subunit protein eL24)	4506619	RPL24	0.0075	1.3	4.5	0.28
77	Coatomer subunit beta (beta-coat protein) (beta-COP)	7705369	COPB1 COPB MSTP026	0.0029	1.5	5.5	0.27
78	Glutamyl-prolyl-tRNA synthetase, isoform CRA_a	119613715	EPRS	0.0007	1.8	6.8	0.26
79	cDNA FLJ61136, highly similar to Ras-related protein Rab-11A	194387154		0.00073	1.3	5.8	0.22
80	Unnamed protein product	34526163		0.0019	1.0	4.8	0.21
81	Dolichyl-diphosphooligosaccharide-protein glycosyltransferase subunit 1	14124942		0.0019	1.0	4.8	0.21
82	Beta-actin-like protein 2 (Kappa-actin)	63055057	ACTBL2	<0.00010	31.5	156.3	0.20
83	Guanine nucleotide-binding protein (G protein), beta polypeptide 2-like 1, isoform CRA_	119574081	GNB2L1	<0.00010	4.5	23.3	0.19
84	Hypoxia up-regulated protein 1 (150 kDa oxygen-regulated protein) (ORP-150) (170 kDa glucose-regulated protein) (GRP-170)	62897071	HYOU1 GRP170 ORP150	<0.00010	4.0	21.3	0.19
85	ABI gene family member 3 (new molecule including SH3) (nesh)	12052938	ABI3 NESH	0.0031	0.8	4.0	0.19
86	Activator of 90 kDa heat shock protein ATPase homolog 1 (AHA1) (p38)	194374633	AHSA1 C14orf3 HSPC322	0.014	0.5	2.8	0.18
87	Phosphoribosylformylglycinamidine synthase (EC 6.3.5.3) (formylglycinamide ribonucleotide amidotransferase) (formylglycinamide ribotide amidotransferase)	119610474	PFAS hCG_31283	0.0049	0.5	3.3	0.15
88	60S ribosomal protein L13 (breast basic conserved protein 1) (large ribosomal subunit protein eL13)	15431295	RPL13 BBC1 OK/SW-cl.46	<0.00010	1.0	7.8	0.13
89	Glucose phosphate isomerase variant	62088730		0.013	0.3	2.3	0.11
90	BH3-interacting domain death agonist (p22 BID) (BID) [cleaved into: BH3-interacting domain death agonist p15 (p15 BID); BH3-interacting domain death agonist p13 (p13 BID); BH3-interacting domain death agonist p11 (p11 BID)]	159163783	BID	0.0074	0.3	2.5	0.10
91	Cullin-associated NEDD8-dissociated protein 1 (Cullin-associated and neddylation-dissociated protein 1) (TBP-interacting protein of 120 kDa A) (TBP-interacting protein 120A) (p120 CAND1)	34782987	CAND1 KIAA0829 TIP120 TIP120A	<0.00010	1.0	13.0	0.08
92	Tripeptidyl-peptidase 1 (TPP-1) (EC 3.4.14.9) (cell growth-inhibiting gene 1 protein) (Lysosomal pepstatin-insensitive protease) (LPIC) (tripeptidyl aminopeptidase) (tripeptidyl-peptidase I) (TPP-I)	15928808	TPP1 CLN2 GIG1 UNQ267/PRO304	0.00069	0.3	3.5	0.07
93	cDNA FLJ54564, highly similar to 150 kDa oxygen-regulated protein (Orp150)	221043650		<0.00010	0.3	5.5	0.05
94	Amyloid beta (A4) precursor protein-binding, family B, member 1 interacting protein, isoform CRA_	119606501	APBB1I	0.0094	0.0	1.8	−inf
95	NEDD8 (neddylin) (neural precursor cell expressed developmentally down-regulated protein 8) (NEDD-8) (ubiquitin-like protein Nedd8)	208435631	NEDD8	0.0094	0.0	1.8	−inf
96	PREDICTED: rho GTPase-activating protein 17 isoform X5 [Homo sapiens]	767988626		0.0094	0.0	1.8	−inf
97	Apoptosis inhibitor 5 (API-5) (anti apoptosis clone 11 protein) (AAC-11) (cell migration-inducing gene 8 protein) (fibroblast growth factor 2-interacting factor) (FIF) (protein XAGL)	377656459	API5 MIG8	0.0048	0.0	2.0	−inf
98	Eukaryotic translation initiation factor 4 gamma 1 (eIF-4-gamma 1) (eIF-4G 1) (eIF-4G1) (p220)	119598670	EIF4G1 EIF4F EIF4G EIF4GI	0.0048	0.0	2.0	−inf
99	Xaa-Pro dipeptidase (X-Pro dipeptidase) (EC 3.4.13.9) (endopeptidase) (peptidase D) (proline dipeptidase) (prolidase)	112491419	PEPD PRD	0.0025	0.0	2.3	−inf
100	RNA transcription, translation and transport factor protein (CLE7 homolog) (CLE) (hCLE)	7706322	RTRAF C14orf166 CGI-99	0.0025	0.0	2.3	−inf
101	cDNA FLJ55804, highly similar to COP9 signalosome complex subunit 1	194388460		0.0025	0.0	2.3	−inf
102	40S ribosomal protein S20 (small ribosomal subunit protein uS10)	226246671	RPS20	0.0013	0.0	2.5	−inf
103	cDNA FLJ56765, highly similar to galactokinase (cDNA, FLJ79080, highly similar to galactokinase)	194383498		0.0013	0.0	2.5	−inf
104	Trifunctional purine biosynthetic protein adenosine-3 [Includes: Phosphoribosylamine--glycine ligase (EC 6.3.4.13) (Glycinamide ribonucleotide synthetase) (GARS) (Phosphoribosylglycinamide synthetase); phosphoribosylformylglycinamidine cyclo-ligase (EC 6.3.3.1) (AIR synthase) (AIRS) (phosphoribosyl-aminoimidazole synthetase); phosphoribosylglycinamide formyltransferase (EC 2.1.2.2) (5’-phosphoribosylglycinamide transformylase) (GAR transformylase) (GART)]	4503915	GART PGFT PRGS	0.0013	0.0	2.5	−inf
105	ADP-ribosylation factor 4	4502205	ARF4 ARF2	0.00065	0.0	2.8	−inf
106	Aspartate--tRNA ligase, cytoplasmic (EC 6.1.1.12) (aspartyl-tRNA synthetase) (AspRS) (cell proliferation-inducing gene 40 protein)	499142118	DARS1 DARS PIG40	0.00033	0.0	3.0	−inf
107	Mitochondrial-processing peptidase subunit alpha (alpha-MPP) (inactive zinc metalloprotease alpha) (P-55)	545478969	PMPCA INPP5E KIAA0123 MPPA	0.00033	0.0	3.0	−inf
108	Nucleosome assembly protein 1-like 1, isoform CRA_b	119617721	NAP1L1 hCG_2015037	0.00033	0.0	3.0	−inf
109	60S ribosomal protein L23	13097600		0.00033	0.0	3.0	−inf
110	COP9 constitutive photomorphogenic homolog subunit 2 (Arabidopsis), isoform CRA_a	119597770	COPS2	0.00017	0.0	3.3	−inf
111	Glutamine--fructose-6-phosphate aminotransferase [isomerizing] 1 (EC 2.6.1.16) (D-fructose-6-phosphate amidotransferase 1) (Glutamine:fructose-6-phosphate amidotransferase 1) (GFAT 1) (GFAT1) (hexose phosphate aminotransferase 1)	183082	GFPT1 GFAT GFPT	0.00017	0.0	3.3	−inf
112	Protein diaphanous homolog 1 (cDNA FLJ61549, highly similar to protein diaphanous homolog 1)	194385544	DIAPH1	<0.00010	0.0	3.5	−inf
113	Phosphoribosyl pyrophosphate synthetase-associated protein 2, isoform CRA_	119570819	PRPSAP2	<0.00010	0.0	4.5	−inf
114	Thymopoietin isoform X	530400796	TMPO	<0.00010	0.0	7.3	−inf
115	Tubulin alpha chain-like 3	13376181	TUBAL3	<0.00010	0.0	7.8	−inf
116	60S ribosomal protein L13 (breast basic conserved protein 1) (large ribosomal subunit protein eL13)	29383	RPL13 BBC1 OK/SW-cl.46	<0.00010	0.0	8.0	−inf
117	Myosin light polypeptide 6 (myosin, light polypeptide 6, alkali, smooth muscle and non-muscle, isoform CRA_c)	119617307	MYL6 hCG_2039617	<0.00010	0.0	9.8	−inf
118	Myl6 protein (myosin, light polypeptide 6, alkali, smooth muscle and non-muscle)	33620739	Myl6	<0.00010	0.0	10.0	−inf
119	Interferon-induced GTP-binding protein Mx1 (interferon-induced protein p78) (IFI-78K) (interferon-regulated resistance GTP-binding protein MxA) (myxoma resistance protein 1) (myxovirus resistance protein 1) [cleaved into: interferon-induced GTP-binding protein Mx1, N-terminally processed]	544711185	MX1	<0.00010	0.0	21.3	−inf
120	MHC class I antigen	350281590	HLA-A	<0.00010	0.0	26.3	−inf
	*Accession number gi-NCBI genInfo Identifer (https://www.ncbi.nlm.nih.gov/protein, accessed on 15 September 2022)						

**Table 2:** Signaling pathways found dysregulated in our study.

	Ingenuity Canonical Pathways *	−log(*p*-Value)	Ratio
1	EIF2 signaling *	13.5	0.065
2	Antigen presentation pathway *	9.7	0.179
3	Crosstalk between dendritic cells and natural killer cells	7.0	0.076
4	Virus entry via endocytic pathways	6.5	0.064
5	Caveolar-mediated endocytosis signaling	6.1	0.078
6	RAN signaling *	6.0	0.211
7	B cell development	5.9	0.109
8	IL-4 signaling	5.6	0.063
9	PD-1, PD-L1 cancer immunotherapy pathway	5.3	0.056
10	Th1 pathway	4.9	0.049
11	Protein ubiquitination pathway *	4.7	0.029
12	Multiple sclerosis signaling pathway	4.3	0.030
13	Phagosome maturation	4.2	0.036
14	FAT10 signaling pathway	4.0	0.068
15	ILK signaling	3.7	0.030
16	Coronavirus pathogenesis pathway	3.7	0.029
17	Th2 pathway	3.6	0.037
18	Interferon signaling *	3.4	0.083
19	Neuroinflammation signaling pathway	3.4	0.021
20	Actin cytoskeleton signaling *	3.3	0.024

* Pathways reported by published studies to be modified in ALS.

## Data Availability

Proteomics data are available via ProteomeXchange with identifier PXD040240.
